# From principles to practice: distributive justice and the role of perceived inequality in reward allocation

**DOI:** 10.3389/fsoc.2025.1660806

**Published:** 2025-12-11

**Authors:** Sandra Gilgen, Christoph Zangger

**Affiliations:** 1 Department of Sociology, University of Zurich, Zurich, Switzerland; 2 Institute of Educational Science, Department of Sociology of Education, University of Bern, Bern, Switzerland; 3 Institute of Applied Data Science and Finance, Business School, Bern University of Applied Sciences, Bern, Switzerland

**Keywords:** distributional survey experiment, distributive justice, merit, need, equality, perceptions of inequality, Gini index

## Abstract

How do people balance competing principles of distributive justice when allocating limited goods? This study applies a novel methodological approach—distributional survey experiments (DSEs)—to examine how people weigh merit, need and equality considerations when deciding how to distribute salaries to people with different ascriptive characteristics and occupations. In the DSE, that we embedded in a representative Swiss survey, respondents were asked to allocate a fixed sum among three hypothetical hospital employees with experimentally varying attributes. This design not only allows us to identify causal factors behind allocation decisions but also captures the underlying interdependence involved in questions of distributive justice. What is more, we can examine drivers of the resulting inequality directly through the allocated salaries. The analyses reveal that although merit-based considerations (occupation, job dedication) have the strongest influence on allocation decisions in the workplace context, need (e.g., having dependent children) and discrimination (e.g., against women and ethnic minority men) also shape outcomes. Moreover, respondents who perceive actual income inequality as too high distribute resources more equally. Conversely, higher-income and higher-status respondents produce more unequal distributions. Our findings highlight how distributional preferences are shaped by justice principles, social background, and inequality perceptions. The study contributes methodologically and substantively to justice and inequality research by linking normative principles and experienced conditions to actual allocation behavior and the distributional consequences thereof.

## 1 Introduction

How do people come up with a fair distribution when the goods to be distributed are limited? Existing research on distributional justice has tried to answer this question by either collecting and analyzing large scale survey data, by means of laboratory experiments with distributional tasks, or through factorial survey experiments in which respondents are presented with, for example, the earnings of different hypothetical people (Alves and Rossi, 1978; Auspurg et al., 2017; Liebe et al., 2020). Each of these approaches comes with both advantages as well as disadvantages in terms of generalizability of the results or the identification of causal mechanisms.

Following different philosophical schools, existing research has identified different distributional principles to which people refer when deciding what they conceive as fair. Some of the most common ones include merit, need, and (strict) equality (Deutsch, 1975; Gilgen, 2022; Lamont, 2017; Miller, 1992). We build on this categorization to investigate how people's allocation decisions in a workplace setting are shaped by these principles as well as (unconscious) biases. We also test whether there is observable heterogeneity by socio-economic background. Additionally, we look at how these distributional preferences, and especially the resulting inequality, are shaped by people's perception of existing economic inequalities.

To do so, we present a new approach to studying distributive justice that combines the advantages of distributional tasks, usually part of laboratory experiments that are often conducted with convenience samples of very specific subgroups of the population (e.g., Akbaş et al., 2019; Hoffman and Spitzer, 1985) with those of survey experiments (e.g., Auspurg et al., 2017; Liebe et al., 2020) embedded within more representative general population surveys. To this end, we developed a distributional survey experiment (DSE; Gilgen, 2022) in which people have to allocate a given sum between three employees of a hospital described in vignettes. Our approach comes with several advantages: As in factorial survey experiments, people are presented with vignettes. In a DSE, however, these are arranged in sets, using efficient algorithms analogous to the construction of discrete choice experiments (Kuhfeld, 2010). A new element of the DSE is that people are not asked to rate the fairness of, for example, earnings of the people described in the form of vignettes, but to actively distribute a fixed amount of money among the vignettes in a given set in a way they deem fair. Doing so allows us to directly examine the underlying preferences and decision-making process since people are forced to weigh up different factors and justice principles simultaneously. Moreover, such a setup directly takes the interdependence of different allocations into account: Since the sum to distribute is fixed, someone's gains will be another's loss, forcing respondents to carefully consider not just the effects of their allocations on one recipient but on the whole group. This makes the data generating process closer to a real-life situation in which scarce goods are distributed among people in different situations and with unique sets of factors that could make them seem more or less needy and/or deserving to the person deciding who gets what.

What is more, the experimental setup of a DSE offers greater flexibility and possibilities in terms of modeling the outcome variable since a monetary value is assigned to each vignette instead of a rating on a fairness scale as commonly used in factorial survey experiments concerned with income inequalities (e.g., Alves and Rossi, 1978; Auspurg et al., 2017; Liebe et al., 2020). In addition to the metric nature and the inherent meaning of the outcome, this is especially useful since it allows us to directly quantify the resulting inequality from people's distributive preferences: Since every respondent distributes a fixed sum across a set of vignettes, we can deduce whose distributions result in greater or lesser inequality.

The contribution of this paper is thus twofold. On the one hand, it introduces a novel and unique experimental approach to studying people's preferences regarding distributive justice and how this translates into overall levels of inequality. On the other hand, it advances the literature on distributive justice by allowing insights into how people balance different justice principles that, given the embeddedness of the experiment in a representative survey, allows for generalization beyond the narrow scope of traditional, often game theoretic, lab experiments. Additionally, by investigating how people's evaluation of inequality—measured as the ratio between ideal and perceived inequality—shapes distributional preferences, we simultaneously examine the impact of both normative beliefs (principles of distributive justice they adhere to) as well as perceived real world conditions on people's allocation decisions.

## 2 Background

Empirical research on distributive justice aims to answer the question what people deem a fair allocation of resources and why. There are many different factors that potentially shape people's views on distributive justice, such as need, merit, equality, efficiency considerations or reciprocity (Gilgen, 2022; Leeds, 1963; Mill, 2008). However, among the many theoretical formulations, three core principles have more or less been established as the most significant for people's justice perceptions: need, equality, and merit (Deutsch, 1975; Miller, 1992). While not fully exhaustive of all the factors that drive people's distributive justice preferences, these three overreaching principles cover most ground while also being maximally independent (Gilgen, 2022).

The principle of need advocates that resources should be allocated based on individual requirements, prioritizing those who have least of any given good or service under consideration. Underlying this principle are humanitarian and social responsibility norms and it is often applied when the goal is to ensure basic wellbeing or survival (Leeds, 1963; Schwartz, 1975). The equality principle, grounded in moral egalitarianism, promotes equal distribution regardless of circumstances—at least in the sense of strict egalitarianism. It is particularly relevant in settings emphasizing solidarity, mutual respect, and harmonious relations (Deutsch, 1975). The merit principle, here used as synonymous to deservingness or proportionality, suggests that distributions among people of a given group or society should be proportional to their contributions. Rooted in notions of equity as fairness (output matches input), it is prevalent in market-based and performance-driven environments, such as modern workplaces or the education system (Adams, 1965; Deutsch, 1975).

Importantly, these principles are not mutually exclusive and can lead to the same distributional outcomes, or conflict with one another (Gilgen, 2022). For example, a high-performing but wealthy individual might deserve a reward based on merit, while a low-performing but needy person may invoke a claim based on need. In such cases, people weigh and prioritize principles based on contextual and/or situational relevance, perceived legitimacy, and expected outcomes (Leventhal, 1976).

At the workplace, organizations typically emphasize merit-based allocations to incentivize productivity and reward performance, be it in terms of promotion decisions or salaries (Cropanzano et al., 2007; Castilla and Ranganathan, 2020). This approach aligns with the broader values of capitalist economies and meritocratic ideals, where fairness is often equated with proportionality of performance and rewards. However, even within the workplace, the application of the merit principle is not absolute: Equality-based distributions may, for example, be adopted in collaborative environments to foster cooperation and trust (Deutsch, 1975), while needs-based considerations may emerge in decisions around employee support programs, parental leave, or accessibility accommodations (Bosch, 2024). Nevertheless, as summarized by (Narisada et al., 2021), merit-based allocations are more generally regarded as fair as compared to needs- and especially equality-based ones in the workplace context.

Preferences for distributive justice are also shaped by individual and social factors, such as people's socio-economic position or (unconscious) biases. Fong (2001), for example, shows that people in lower socio-economic positions are more supportive of redistributive policies, aligning with the need principle. People who primarily attribute success to effort rather than luck are more likely to endorse merit-based distributions (Fong et al., 2006). Women have been found to show more egalitarian and needs-oriented preferences, which is often attributed to gender-specific socialization and the experience of systematic disadvantage, while men react stronger to competition (Kivikangas et al., 2014) and therefore show stronger preferences for a merit-based approach (Gilgen, 2022).

While equality-based distributions, such as a free parking space for all employees, can increase employees' satisfaction and are largely regarded as fair (Cropanzano et al., 2007), people often expect that monetary “rewards”, that is, salaries, are distributed more or less proportionally to contributions (Liebig et al., 2015; Narisada et al., 2021). Thus, when it comes to the wages of employees, even though the extent to which merit considerations guide salary allocations likely differs with characteristics of the decision maker (e.g., gender, own social position) as well as characteristics of the recipient (e.g., how “needy” they are), we can expect merit to be the guiding underlying principle of distributive justice. While observational as well as experimental studies suggest that people consider additional principles, especially need, as well as ascriptive characteristics such as gender or age, even in work-related situations (Auspurg et al., 2017; Gilgen, 2022). On the one hand, the prevalence of additional principles of distributive justice can be explained by the beneficial impact they have on long-term cooperation within organizations, for example through the reciprocity norm (Ostrom, 1998). On the other hand, alternative mechanisms, such as ingroup favoritism or system-justifying processes, might also lead to similar outcomes and make it difficult to disentangle from the principles of distributive justice (e.g., when people reward different professional qualifications based on social status rather than the underlying human capital and skills; Jost et al., 2004; Horwitz et al., 2014; Mattan et al., 2019).

### 2.1 Perceived inequality and distributional justice

Social contexts matter for people's attitudes toward distributive justice (Gilgen, 2022). While the perceived legitimacy of principles of distributive justice vary across societies (e.g., a higher prevalence of merit-based considerations over, for example, need in the US context; Janmaat, 2013), there is also, as mentioned above, heterogeneity within countries, for example between different socio-economic groups (Fong, 2001; Fong et al., 2006). One important contextual factor influencing what principles of distributive justice are applied is the (perceived) level of inequality within a society or more globally. A possible mechanism is that inequality has different implications on people's well-being and life chances depending on their own social position, which in turn can affect their level of politicization and willingness to engage in collective action (e.g., Huang, 2019; Petkanopoulou et al., 2025).

Existing studies linking (perceived) inequality and views on distributive justice or fairness of earnings often refer to the concept of existential standards. Existential standards—as opposed to utopian standards, which would correspond more closely with normative views on principles of distributive justice—refer to the observation that people often use a set of observed, established practices (the “what is”) to obtain a conception or a judgment on whether something is fair or just (the “what ought to be”; Shepelak and Alwin, 1986). That is, people derive their notions of what is considered “just” by observing and internalizing the “going rates of return” to various characteristics in the real world (Shepelak and Alwin, 1986). In the context of inequality, people conceive a distribution or allocation of rewards and resources as unjust, if it deviates from established practices: “If I have the same skill level and experience, I should get the same salary as my coworker in the same position. If they get more, I will conclude that I am unfairly underpaid”. This very simple conceptualization has led some authors to argue that we can adequately capture people's conceptions of distributive justice by measuring the discrepancy between what—in people's views—is, and what ought to be. Jasso's (1978) justice evaluation function, which is the logarithm of the ratio of actual and just earnings, both assessed subjectively by the individual, relies on this logic.

Existing research indeed finds evidence for the intertwined impact of perceived inequality and people's fairness evaluations and the willingness to take action to address inequalities. Huang (2019) shows for China how personal beliefs on distributive justice beliefs—an individual's perception of whether they receive what they deserve—mediates the impact of inequality on happiness. This finding aligns well with the work by Shepelak and Alwin (1986) who find in their vignette study that people indeed “take the way things are” as a basis to judge the fairness of their distribution of rewards. Similarly, Petkanopoulou et al. (2025) recently showed in a series of experiments how perceptions of inequality are linked to the willingness to take collective action. Therein, again, views on distributive justice take a mediating role. Nevertheless, as Heiserman and Simpson (2021) note, the link between justice perceptions and attitudes toward inequality is not straightforward and different factors can suppress people's negative emotional reactions to deviations from the desired state.

Although measuring distributive justice in terms of the deviation of “what is” from “what ought to be” (Jasso, 1978; Shepelak and Alwin, 1986) is intriguing, it builds on the assumption that the underlying principle which is observed in practice is universally accepted as just. However, even if, for example, rewards are completely allocated in accordance with the merit principle in a given firm, and all the people in this firm acknowledge merit as the most legitimate principle of distributive justice in this setting, a person might nevertheless individually prefer allocations according to need. If this is the case, the whole notion of existential standards (the observed practices of reward allocation in a society) does not actually capture “what ought to be”. Shepelak and Alwin (1986) also note in their study, that only about a third of the variation in justice beliefs represent shared aggregate principles. Consequently, we need to complement such measures of distributive justice with a broader view on the underlying principles when studying how people's allocation decisions are affected by perceived inequality.

### 2.2 The present study

Existing research on distributive justice usually focuses on the fairness of given allocations (Auspurg et al., 2017; Jasso and Rossi, 1977; Liebe et al., 2020). While this helps us understand *what* people consider a just allocation of resources, *how* the underlying principles shape actual allocation decisions and the resulting (in)equality remain largely unanswered by this line of research. On the other hand, experimental studies that focus directly on allocation tasks often face problems of external validity. What is more, studies focusing on the impact of perceived inequality largely fail to explicitly account for normative principles of distributive justice in a causal way since they usually operationalize distributive justice in terms of the difference between what people think is a just salary and what people earn (Jasso, 1978; Shepelak and Alwin, 1986).

Against this background, we use a novel approach, a distributive survey experiment (DSE), described in more detail below, to show how people can be guided by different principles of justice (merit, need, equality) simultaneously when distributing limited goods. The DSE also allows us to observe who attaches more importance to which principle. In a next step, we examine the extent to which we can explain the resulting inequality from the distribution of goods/wages in the experiment by people's perceptions of existing economic inequality, measured as the ratio of desired vs. estimated actual earnings for low- vs. high-skilled workers, as well as how these distributions differ between socio-economic groups. This approach deviates from simple, individualistic assessments of just earnings that only compare people's actual salaries with what they think they should earn and offers a more comprehensive view. Using the DSE thus helps us understand how perceived inequality affects distributional decisions and therewith results in more or less economic inequality. Doing so allows us to evaluate who attaches what weight to the different principles of distributive justice in a workplace setting. The approach also allows us to directly assess the resulting consequences in terms of economic inequality and to understand how the underlying distributional decisions are shaped by perceived levels of inequality.

## 3 Data and methods

### 3.1 Distributional survey experiments

To examine people's preferences for (un)equal earnings distributions, we used a distributional survey experiment (DSE; Gilgen, 2022). In a DSE, respondents are asked to distribute a given sum among hypothetical people described in vignettes. The different characteristics that make up the vignettes are experimentally altered using *D*-efficient fractional factorials and are obtained by first finding a suitable linear arrangement (as in a factorial survey experiment; Auspurg and Hinz, 2015), which is then efficiently allocated to different sets, using the algorithms to design discrete choice experiments (Kuhfeld, 2010). While the outcome in a discrete choice experiment is categorical (choice of one of several alternatives), DSEs, like factorial survey experiments, have outcomes on a metric measurement level. More importantly, the outcomes of a given set of hypothetical vignette people are interdependent: Someone's gain is another's loss—reflecting real-world distributional dilemmas in the case of finite resources.

Consequently, DSEs combine allocation tasks common in laboratory experiments on distributive preferences (e.g., Kittel et al., 2020; Levitt and List, 2007) with the complex design of discrete choice experiments that allow for the causal investigation of multiple treatments applied to representative samples of the population (Gilgen, 2022). Existing studies using factorial survey experiments evaluate people's fairness perceptions of given (un)equal distributions (e.g., of earnings; Auspurg et al., 2017; Jasso and Rossi, 1977; Liebe et al., 2020). They thus do not directly answer questions about how distributional preferences shape (unequal) allocations but examine to what extent the resulting distributions are perceived as fair. DSEs allow us to directly examine the underlying allocation decisions that give rise to these (un)equal distributions in the first place. As a consequence, they are especially useful for the causal assessment of people's distributional preferences as well as the resulting inequality.

### 3.2 Data

For this study, we draw on a DSE that was implemented in the 2019 MOSAICH survey in Switzerland (Ernst Stähli et al., 2020). MOSAICH is a cross-sectional survey that is conducted every 2 years as a Swiss add-on to the International Social Survey Program (ISSP). Thematically, it evolves around the corresponding rotating module of the ISSP, supplemented by additional modules for the Swiss context. The present DSE was implemented in the 2019 round following an open call for survey items in a module focusing on social inequalities.

#### 3.2.1 Measures in the distributional survey experiment

In the DSE, respondents were asked to put themselves in the situation where they had to freely distribute a fixed amount of CHF 18,000 (approx. $ 20,500) among three new employees who all worked full-time in a hospital. They were, however, not explicitly primed to imagine themselves in the position of a hospital executive who has to make managerial decisions. Instead, we wanted the respondents to make distributions in line with their own concept of distributive justice. The hospital setting was deliberately chosen since people have an approximate idea about different positions and professions within a hospital. The three employees were described in vignettes, in which the characteristics, summarized in Table 1, were experimentally manipulated. Following the suggested number of experimental treatments in the literature (Auspurg and Hinz, 2015), a total of 7 characteristics was manipulated in order to assess which principles of distributive justice—need, merit and equality (Deutsch, 1975; Miller, 1992)—were applied, as well as to test for discrimination by gender and ethnic origin. The chosen characteristics, especially those used to measure merit and need, are based on measures in Gilgen (2022), who used both quantitative as well as qualitative pretesting (think aloud and probing) to assess the validity of the different treatments. The hypothetical employees' gender and ethnic background were indicated by their names so that the manipulation was not too obvious (Auspurg et al., 2017; Gilgen, 2022; Gilgen and Stocker, 2022). For each gender, a first and last name was chosen that indicated a specific ethnic background, namely Emma or Daniel Meier for a Swiss background, Jelena or Ilija Nikolic for a Serbo-Croatian-speaking background, and Leila or Amir Mansour for an Arabic-speaking background.

**Table 1:** Experimentally manipulated characteristics and their levels.

**Dimension/attribute**	**Levels/values**
***Ascriptive***	
Gender	Man; woman
Background	Swiss name; Serbo-Croatian name; Arabic name
***Need***	
Partner	Single/single parent; has a partner
Kids	No kids; 2 children
Health	In good health; in poor health
***Merit***	
Occupation	Cleaner; nurse; medical doctor
Job commitment	Very committed; sometimes more, sometimes less committed; not very committed

Need was operationalized by experimentally manipulating the described employee's family status (single/single parent vs. with partner), whether they have children (no children vs. 2 children), and their health status (in good health vs. in poor health). While the first two measures refer to need in terms of people who are co-dependent on the vignette person's income (children) or who can support said person (partner), the latter more directly measures the need of people depicted in the vignettes. Previous research has used the same measures (health, children; Auspurg et al., 2017; Liebig et al., 2015) to assess need and all three of them, including family status, have been qualitatively pretested in other work (Gilgen, 2022) to make sure people interpret these dimensions in the intended form and not, for example, as an additional performance indicator (e.g., diminished work capacity due to poor health).

Merit was measured by experimentally varying two characteristics in the vignettes. On the one hand, we randomly altered the profession of the employees described in the vignettes, differentiating among a cleaner, a nurse, and a physician who all work in the same hospital. The three professions reflect different qualifications and educational pathways, mirroring varying amounts of invested human capital. This is why occupation is often taken as a proxy for varying skill levels in the literature on wage regression (Levenson and Zoghi, 2010). Since it can be expected that respondents have an approximate idea of what a cleaner, a nurse, and a medical doctor do, these three professions were chosen. However, occupations might not only signal differences in invested human capital and skills, but also reflect different social positions. It is thus possible that the effects of occupation also reflect the principle of entitlement rather than just merit (Liebig et al., 2015). Furthermore, employees' commitment to their job was also experimentally manipulated. Referring to their last certificate of employment, they were either described as very committed, sometimes more, sometimes less committed, or as not very committed. Equality preferences are not directly operationalized in the DSE. Instead, we infer those preferences from people's distributions among the three vignette individuals described in a set.

Together, these seven characteristics with two or three levels each yield a vignette universe (full factorial) of 2^4^×3^3^ = 432 unique treatment combinations. This full factorial was reduced to a *D*-efficient fractional factorial of 72 unique vignettes using algorithmic searches. The *D*-efficiency of the linear arrangement, which is the basis for the description of the employees in the experiment, was 98.9. These vignettes were then efficiently allocated to 24 different choice sets. The final setup of this allocation has a relative *D*-efficiency of 94. In the design, all two-way interactions are identifiable. One out of the 24 different choice sets was randomly allocated to each respondent as part of the add-on survey module. Based on the information presented in the experiment, they then had to distribute a total of 18,000 Swiss Francs among the three vignette people.

#### 3.2.2 Additional covariates

Since this DSE was implemented in a representative, cross-sectional survey, we have rich information on the respondents. Furthermore, the DSE was part of a larger module on social inequality in which, among other things, respondents also assessed the income of people working in different professions, including a medical doctor and an unskilled worker, and they were also asked what they think these people should earn. These four measures allow us to calculate the perceived as well as desired income inequality for these two professions by dividing the estimated earnings of a medical doctor by the estimated earnings of an unskilled worker for each respondent. Likewise, calculating the same ratio for what people in the two professions should earn allows us to measure respondents' ideal level of income inequality. Finally, we build on Jasso's (1978) measure of distributional justice and divide the perceived by the desired level of inequality. This is in line with Shepelak and Alwin's (1986) idea of assessing the existential dimension of distributional justice and reflects the extent to which people evaluate the underlying income inequality as justified, unjustly low (values smaller than 1) or unjustly high (values above 1).

Moreover, we account for heterogeneous preferences and test for in-group favoritism by taking a variety of respondent background characteristics into account. Namely, we include respondents' gender, age, migration background, subjective class position, and personal income to test whether there are differences in distributional preferences by socio-economic background and whether people tend to allocate more to people who are more similar to themselves. Table A1 in the appendix summarizes the descriptive statistics for these as well as the manipulated covariates in the experiment.

### 3.3 Analytical strategy

Although a DSE is set up like a discrete choice experiment, the outcome is a metric variable for each of the different alternatives. What is more, the outcome directly reflects the interdependence inherent to the distributional task: One person's gain is another's loss. This highlights the hierarchical nature of the data: Each respondent is responsible for three outcomes, that is, the salaries distributed to the three vignette people. While we would commonly accommodate this structure by means of a random or fixed effects model (Hox et al., 2017), the variance at the respondent level is fixed due to the distribution of a limited amount of CHF 18,000 among the three vignettes. We therefore account for the hierarchical structure of the data by means of clustered standard errors instead (Cameron and Miller, 2015).

For the present purpose, we estimate two different linear models. In the first one, we regress the amount of money a vignette person receives, *y*_*i*_, on the characteristics manipulated in the experiment, *D*_*i*_, as well as the respondent-level covariates, *X*_*i*_:


$$\begin{array}{l} y_{i}=\delta^{T}D_{i}+\beta^{T}X_{i}+\varepsilon_{i}, \end{array}$$ (1)

Where, **δ**^**T**^ is a row vector of treatment effects, **β**^**T**^ is a row vector for the coefficients of the respondent-level covariates, and ε_*i*_ are the residuals. Using the traditional Huber-White estimator, the variance for $\hat{\text{δ}}$ is given by


$$\begin{array}{l} Var\left(\hat{\text{δ}}\right)={\left({\text{D}}^{T}\text{D}\right)}^{-1}{\text{D}}^{T}\left[\begin{array}{ccc} {\hat{\sigma }}_{1}^{2} & \cdots \\ \vdots & \ddots & \vdots \\ \cdots & {\hat{\sigma }}_{g}^{2} \end{array}\right]{\text{D}\left({\text{D}}^{T}\text{D}\right)}^{-1}. \end{array}$$ (2)

**Figure 1:**
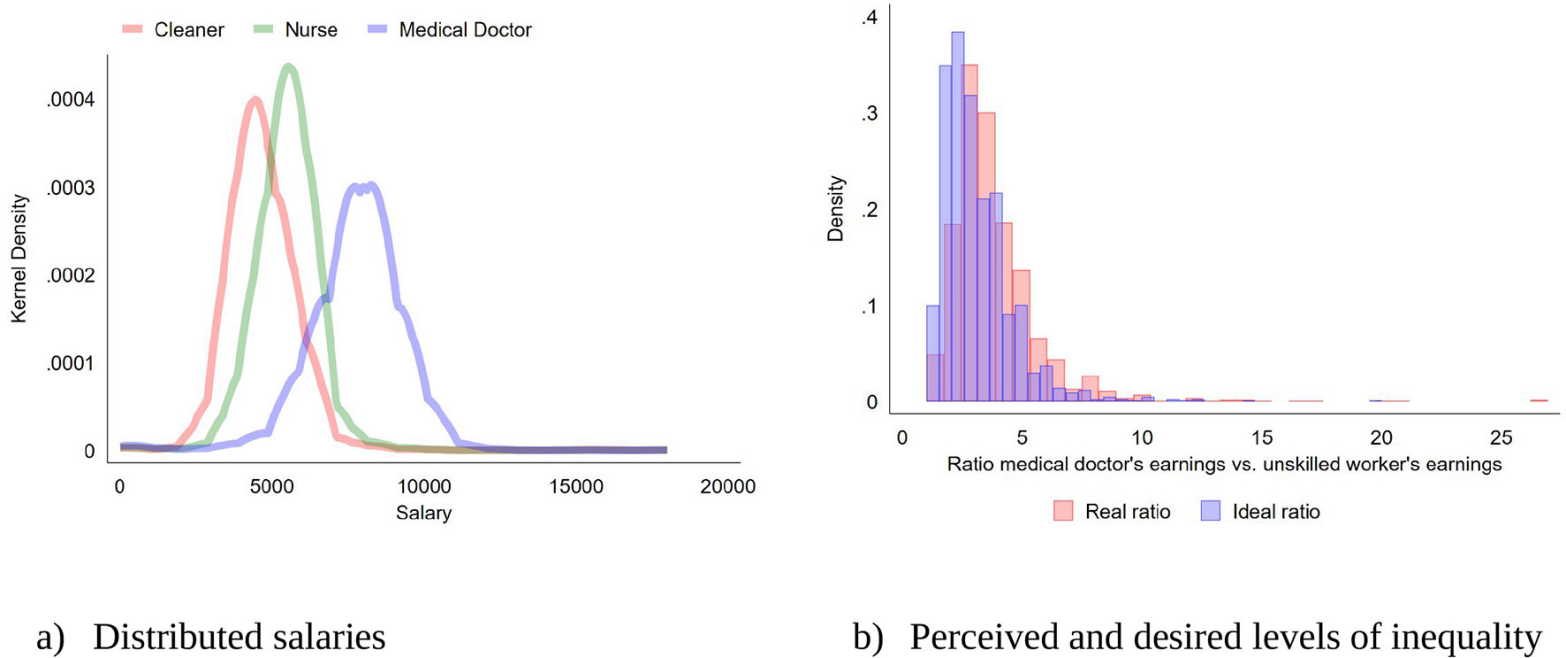
Distribution of the allocated sum to the three different professions as well as people's perceived and desired level of inequality.

To analyze the inequality resulting from the respondents' distributions of money to the three vignette people, we calculate the Gini coefficient of the distributed salary for each respondent separately. As a robustness check, we further also calculate the generalized entropy as well as the Theil index (Cowell, 2000; Shorrocks, 1984). The generalized entropy measure accounts for the presence of very small, rather than very large incomes, setting the corresponding parameter *c* in


$$GE(c)=\{\begin{array}{c} \frac{1}{G\times c(c-1)}\sum_{1=1}^{G}\left[{\left(\frac{y_{i}}{y}\right)}^{c}-1\right],\;c\neq \{0,1\},\\ \frac{1}{G}\sum_{1=1}^{G}\frac{y_{i}}{y}\;ln\left(\frac{y_{i}}{y}\right),\;c=1,\\ -\frac{1}{G}\sum_{i=1}^{G}\;ln(\frac{y_{i}}{y}),\;c=0. \end{array}$$ (3)

to *c* = 0 with *G* = 3, the number of cases within each group, that is, the hypothetical vignette people among whom respondents distributed the CHF 18,000. While the distributions are approximately normal (see Figure 1, panel a), there are a few cases with (relatively speaking) very low salaries, especially in the case of medical doctors. The Theil index is a special case of the generalized entropy measure in Equation 3 with *c* = 1.

After calculating the inequality measures using the original data (vignette level), we then aggregate the data to the respondent level for the second part of our analyses. Doing so allows us to answer the question whose distribution results in greater inequality. To this end, we again estimate a simple linear model, this time omitting the experimentally manipulated characteristics:


$$\begin{array}{l} y_{j}=\beta^{T}X_{j}+\varepsilon_{j}, \end{array}$$ (4)

That is, we predict the observed inequality *y* for each respondent *j* as the main outcome. While we now only have one observation per respondent, there are 24 unique choice sets, that is, combinations of the 72 (3x24) hypothetical employees. When estimating Equation 4, we therefore cluster standard errors at the set level. Finally, since the dependent variable in Equation 4 lies in the domain of $R_{+}^{n}\to \left[0,1\right]$ for the Gini coefficient and $R_{+}^{n}\to \left[0,\infty \right)$ for the generalized entropy measures (although in the data they barely reach values close to 1), we use the log of the ratios depicted in Figure 1b to account for this differential scaling.

In addition to the robustness checks mentioned above (looking at generalized entropy-based measures of inequality), we also conduct a series of additional checks. First, we include the vignette position to check whether respondents relied on heuristics instead of making evaluations of all three hypothetical employees in a given set (e.g., considering only the first two hypothetical people in the set, and giving less or nothing to the last one). Second, we model the distribution of the inequality measures more explicitly by also running Beta regressions (for the Gini coefficient), as well as Generalized Linear Models with a Gamma log-link for the two entropy-based inequality measures. Finally, we use Tobit regression models for all three inequality measures to account for left censoring (people who distribute equally among the three vignettes).

## 4 Results

### 4.1 Distributional preferences

We start our examination of people's distributional preferences by looking at how respondents distributed the total of 18,000 Swiss Francs among the three hypothetical vignette people they were presented in the DSE. In each set, there was a cleaner, a nurse, and a medical doctor working in the same hospital. From Figure 1a, we infer that people differ considerably in how they distributed the total amount among the three hypothetical employees. While all three outcomes are roughly normally distributed, people, on average, give most to a medical doctor and least to a cleaner, although the sums allocated to a cleaner and a nurse overlap considerably.

Furthermore, respondents differ considerably in their perceived as well as ideal inequality levels. In this regard, Figure 1b shows that most people (in contemporary Switzerland) perceive and desire some inequality, measured as the ratio between a medical doctor's and an unskilled worker's salary. Noteworthy, the ideal inequality level is slightly lower than the perceived level of inequality, and there is a minority of individuals who both perceive and aspire for quite high inequality, with ratios exceeding 10. Additionally, while the correlation between perceived and ideal inequality is quite high with 0.56, the two measures are only weakly correlated with respondents' personal income (0.06 and 0.15 for perceived inequality and ideal inequality, respectively).

Looking at what factors people take into consideration when making allocation decisions according to their sense of distributive justice, we find that they not only consider need and merit, but that they also tend to discriminate, especially against men with a Serbo-Croatian or Arabic name and against women. The first model in Table 2 shows the results when only considering the vignette characteristics, while models two and three add (interactions with) respondent-level covariates. Throughout all three models, there is a clear pattern in the effects of the experimentally altered characteristics: People allocate about 300 Swiss Francs more per month if the hypothetical person has children, whereas health status, as a further indicator of need, is considered irrelevant. In terms of merit, respondents reward job dedication with about 385 and 825 Swiss Francs more per month if the employees are described as being either more or less or very dedicated to their job compared to showing low dedication. Likewise, compared to a cleaner in the same hospital, a nurse is allocated about 800 Swiss Francs more, while a medical doctor gets a premium of about 3,150 Swiss Francs per month.

**Table 2:** OLS estimates for the amount distributed among vignettes.

	**Vignette only**		**Respondent characteristics**		**Interactions**	
**Vignette characteristics**						
Gender (*Ref.: Woman*)						
Man	225.7^***^	(59.15)	225.8^***^	(59.17)	217.2^***^	(58.31)
Background (*Ref.: Swiss name*)						
Serbo-Croatian name	−62.02	(53.57)	−61.73	(53.63)	−78.40	(52.70)
Arabic name	−8.871	(56.60)	−8.802	(56.61)	−19.25	(55.81)
Gender × Background						
Man with Serbo-Croatian name	−182.4^*^	(82.57)	−183.0^*^	(82.70)	−162.8^*^	(81.96)
Man with Arabic name	−171.3^*^	(76.37)	−171.5^*^	(76.39)	−167.1^*^	(75.71)
Relationship status (*Ref.: Has partner*)						
No partner	6.688	(36.03)	6.770	(36.03)	7.268	(35.68)
Children (*Ref.: No children*)						
2 children	297.3^***^	(39.51)	297.4^***^	(39.52)	299.1^***^	(39.04)
Health status (*Ref.: Poor health*)						
Healthy	37.60	(37.48)	37.58	(37.48)	35.96	(37.06)
Job (*Ref.: Cleaner*)						
Nurse	796.5^***^	(35.00)	796.5^***^	(35.01)	757.2^***^	(111.5)
Medical doctor	3150.4^***^	(54.81)	3150.4^***^	(54.82)	3175.1^***^	(188.9)
Dedication (*Ref.: Not very dedicated*)						
More or less	385.2^***^	(46.40)	385.3^***^	(46.42)	384.2^***^	(45.52)
Very dedicated	828.9^***^	(47.85)	828.9^***^	(47.86)	830.6^***^	(47.09)
**Respondent characteristics**						
Ratio perceived vs. ideal inequality			−5.851	(6.091)	183.9^***^	(48.25)
Personal income			1.143	(1.679)	−43.62^***^	(10.32)
**Respondent-vignette interactions**						
Job × perceived vs. ideal inequality						
Nurse					−81.72	(58.02)
Medical doctor					−487.3^***^	(102.3)
Job × personal income						
Nurse					25.68	(13.67)
Medical doctor					108.5^***^	(20.92)
*N*	4863		4863		4863	
adj. *R*^2^	0.601		0.601		0.613	

Moreover, respondents also respond to the ascriptive characteristics of the employees described in vignettes to determine their salary. In this respect, they discriminate against women, who, on average, receive about 225 Swiss Francs less per month, as well as against minorities. The significant interaction effect between a vignette person's gender and ethnicity uncovers that the overall negative effect of a Serbo-Croatian or Arabic name compared to those with a Swiss name is completely attributable to minority men. Men with a Serbo-Croatian or Arabic name are paid between 170 and 180 Swiss Francs less per month, while minority women have no additional penalty on top of the strong gender bias.

While adding respondent characteristics in model 2 of Table 2 does not generally change the sum vignette people receive (due to the random allocation of the vignettes and the fixed sum to distribute), the interactions of both the ratio of respondents' perceived and ideal levels of inequality as well as their personal income significantly shape how much money they allocate to hospital employees in different professions (model 3). People who evaluate existing income inequality as much higher than what they would prefer (larger ratios of perceived vs. desired inequality) tend to distribute more equally, taking, on average, about 490 Swiss Francs from what respondents think a medical doctors' salary should be, and giving it to a cleaner in the same hypothetical hospital. Meanwhile, with increasing own personal income, respondents tend to take from the cleaner and give to the medical doctor: With each increase of 1,000 Swiss Francs in personal monthly income, respondents tend to give about 110 Swiss Francs more to the medical doctor compared to the cleaner. This effect could also reflect in-group favoritism rather than more inegalitarian preferences of richer respondents (Jost et al., 2004; Horwitz et al., 2014). However, interacting vignette occupation with respondents' subjective class position reveals few and rather heterogeneous effects (not reported in Table 2): While people in lower social classes do not allocate more money to a cleaner or a nurse, people who identify as upper-middle class, for example, give more to a nurse while people who identify as upper class do not tend to give more to a medical doctor in the experiment. Consequently, in-group favoritism does not seem to explain the income-dependent allocation decisions (Jost et al., 2004). Although also not reported in Table 2, we find some evidence for in-group bias with regard to ethnic background, but not gender: Swiss people tend to give more to people with a traditional Swiss name. Moreover, respondents from Eastern and South-Eastern Europe tend to give more to people with a Serbo-Croatian name, while people with an Arabic name receive more from respondents with a migration background from a non-Western, non-European country.

### 4.2 Resulting inequality

While the aforementioned results reported in Table 2 already point out whose distributional preferences might lead to greater inequality, we further examine this by directly modeling the resulting inequality of respondents' distributions among the three vignette people. To this end, we regress the resulting Gini coefficient on respondents' gender, migration background, subjective class position, income, age and the ratio of their perceived vs. ideal inequality levels. The vignette characteristics are not included in this aggregated data since the attributes are randomly distributed over the vignette sets seen by respondents.

**Figure 2:**
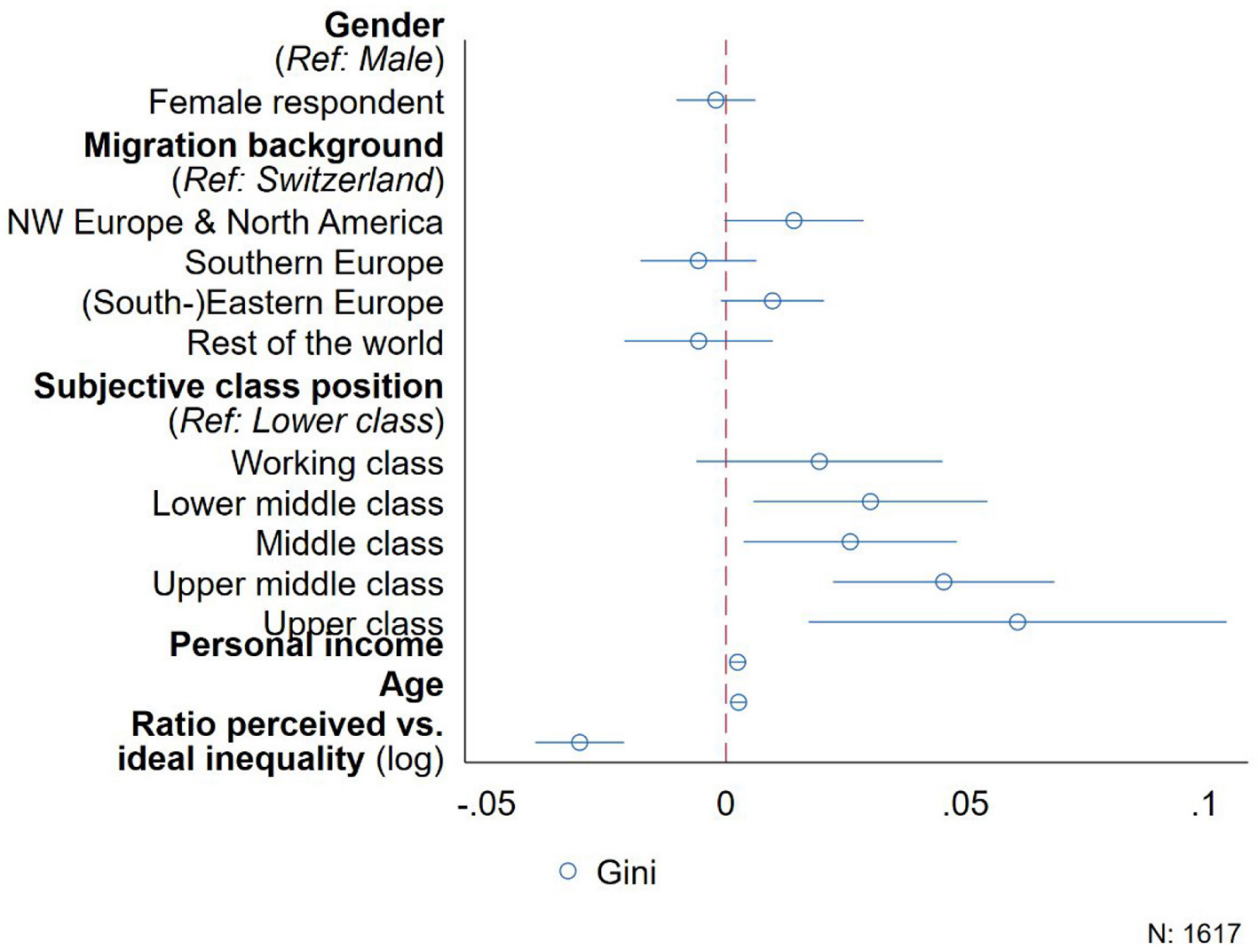
Differences in resulting inequality (Ginicoefficient) by respondent characteristics.

While the results in Table 2 show that respondents discriminated against female employees, Figure 2 shows that male and female respondents do not differ in the extent to which their distributional preferences shape inequality. However, compared to people without a migration background, respondents from North-Western Europe and North America tend to allocate money in such a way that the overall inequality increases. More strikingly, however, people in higher subjective class positions and with higher incomes distribute salaries in such a way that increases inequality considerably, resulting in an up to 0.05 points higher Gini coefficient when comparing people who see themselves as belonging to the upper middle or upper class with those from the lower class. Also, every increase in personal income by 1,000 Swiss Francs per month raises the Gini coefficient of the salary distributions by 0.002 points on average.

What is more, the larger the discrepancy between respondents' perceived and desired level of inequality in contemporary Switzerland, the more equally they distribute salaries among the people in the hypothetical hospital. For every increase in the ratio of perceived and desired level of income inequality, respondents, on average, distribute in such a way that the corresponding Gini-coefficient is reduced by about 0.03 points. Finally, in the data we also find that older respondents tend to allocate salaries more unequally, although this effect is rather small.

### 4.3 Robustness

We perform a series of robustness checks to confirm the overall pattern obtained from the DSE. We first check whether respondents really took all three employees described in the vignettes into consideration when deciding who should get how much. Since the underlying set-up as a choice experiment tries to maximize variance, people are forced to make trade-offs and face potentially hard decisions. Coming up with a fair distribution can therefore be quite a demanding task for respondents. Respondents might therefore have simplified the task by not paying equal attention to all vignettes but, for example, paying more attention to the first and second one while neglecting the third. Although existing research suggests fatigue effects only start setting in at 10 or more vignettes per respondent (Auspurg and Hinz, 2015), we included the vignette position in the regression model in Table 2 as a first robustness check to test whether respondents considered all three vignettes to the same extent. This yielded an insignificant effect, suggesting that respondents considered all three vignettes equally, irrespective of their position in the set.

**Figure 3:**
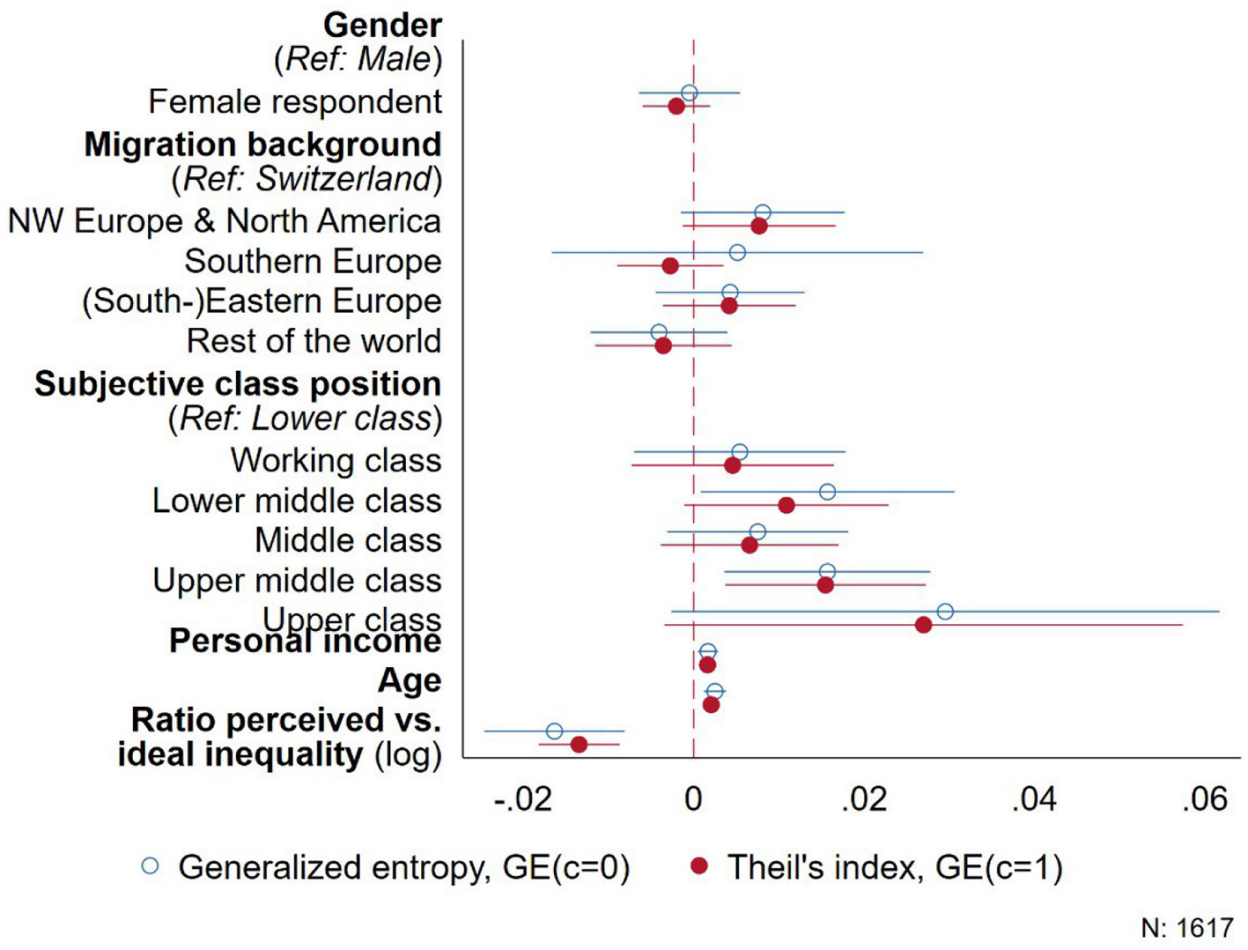
Generalized entropy-based measures of inequality.

Since a single measure is usually ill-suited to summarize a distribution (Liu and Gastwirth, 2020), we evaluated to which extent other inequality measures reproduce the patterns documented in Figure 2 as a second robustness check. In this respect, we calculated two generalized entropy measures for each respondent's distribution among the three vignettes, setting *c*= {0,1} in Equation 3, that is, a measure that accounts for very low salaries (*c* = 0) and the Theil index (*c* = 1). The overall pattern obtained above is reproduced when using these alternative inequality measures, although some of the associations are not statistically significant. From Figure 3, we infer that the distributions of people with higher income as well as older people still result in increased inequality, both overall (Theil index) as well as at the lower end of the distribution (generalized entropy with *c* = 0). Using these alternative measures, only people who see themselves as upper middle class significantly differ from people who describe themselves as lower class, while migration background has no statistically significant effect. The larger standard errors in Figure 3, however, also indicate heterogeneity and small case numbers, for example, for people who declare they are upper class (see Table A1 in the appendix). Consequently, using these additional measures of inequality generally confirms the overall picture from Figure 2, according to which especially older people in more privileged social positions tend to distribute salaries among the hypothetical employees in the experiment in a way that increases overall economic inequality.

**Figure 4:**
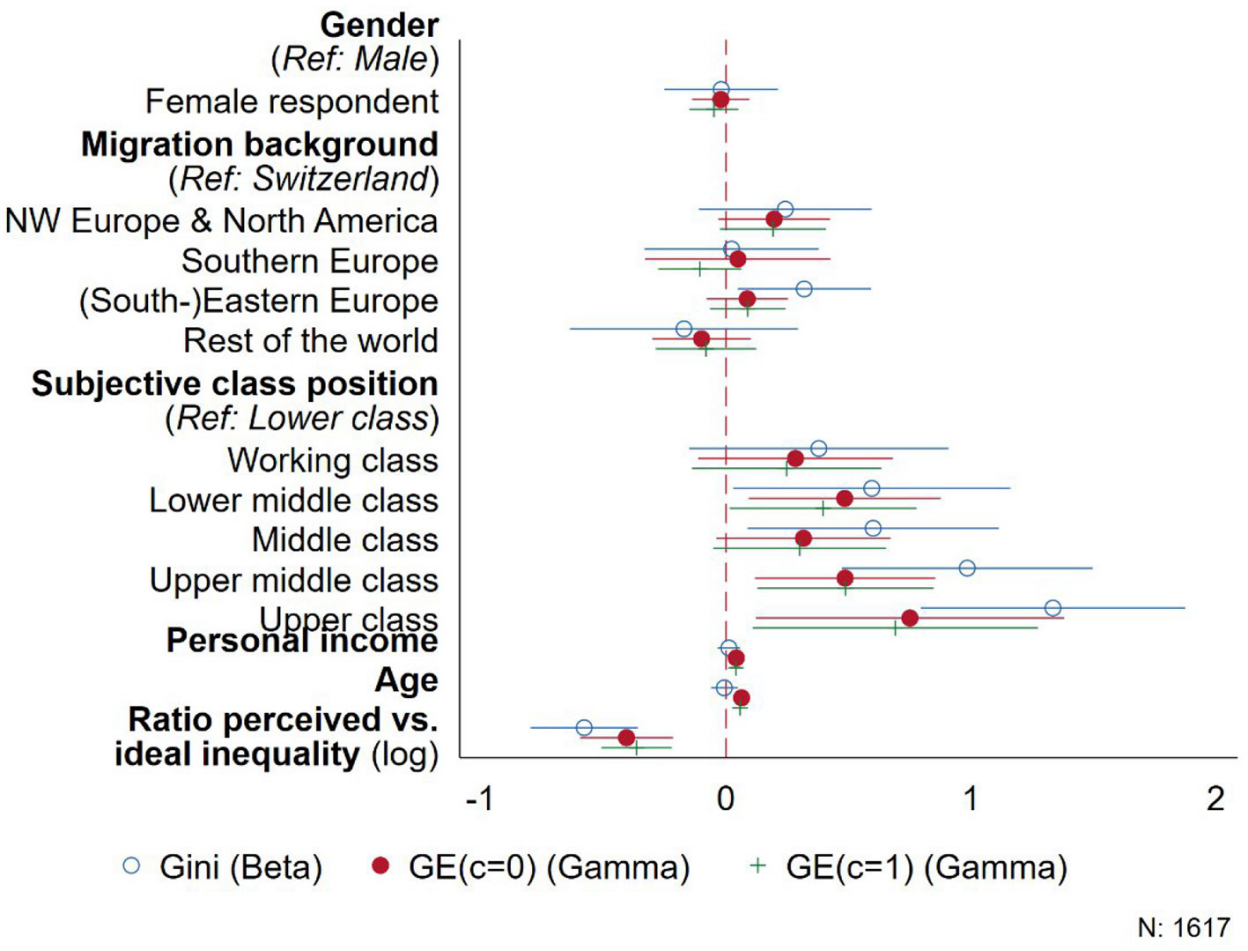
Comparison of Beta (Gini) and Gamma (Generalized entropy measures) regressions.

**Figure 5:**
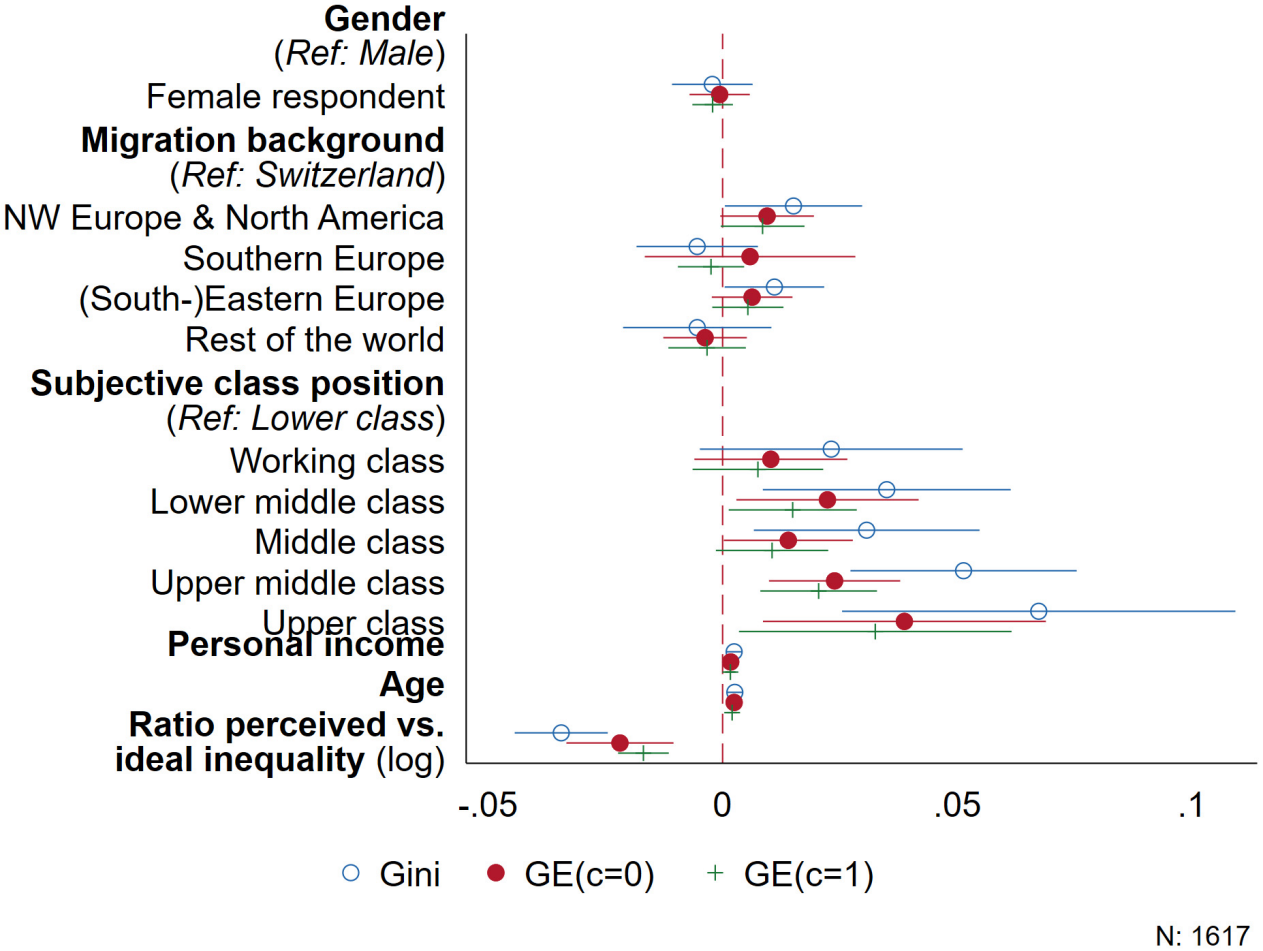
Results from Tobit regression models.

As a third robustness check, we use Beta- and Gamma-regression models, as well as Tobit models to more explicitly account for the lower bound of 0 in all three inequality measures as well as an upper bound of 1 for the Gini coefficient. Figure 4 depicts the results using a Beta regression model for the Gini coefficient, as well as Gamma regressions for the generalized entropy measures with *c* = 0 to account for very small values (i.e., people who distribute equally among the three vignettes) and *c* = 1, the Theil index. Additionally, Figure 5 contains the corresponding results of Tobit regression models for all three inequality measures.

Accounting more explicitly for the bounded nature of the different inequality measures confirms the pattern of effects found before in Figures 2, 3. All six models show a significant association of the inequality measures with people's subjective class position and income on the one hand, as well as the ratio of perceived vs. desired inequality on the other. Again, these effects are generally stronger when using the Gini coefficient, while the two entropy-based measures reveal smaller but often more precise estimates. In contrast to the linear model depicted in Figure 2, using a Beta regression to account for the lower and upper bound of the Gini coefficient results in a coefficient for age that is not statistically significant. However, in the Tobit models in Figure 5, age has a consistent positive correlation with all three inequality measures: Older people tend to distribute salaries among the three employees in a way that increases overall inequality.

Summing up, both the main results as well as the different robustness checks depict a clear picture: people in higher self-attributed class positions and those with higher personal incomes distribute salaries in the distributional survey experiment in such a manner that increases inequality, irrespective of the measure used. Moreover, the distributions of salaries by respondents who perceive a higher level of inequality in the real world compared to what they deem just lower overall levels of inequality. Finally, while men and women do not differ regarding the inequality resulting from their allocations, people with a migration background from North-Western Europe and North America as well as older people distribute in such a way that increases inequality.

## 5 Discussion

This study examined how people balance different principles of distributive justice and what other factors people consider when making allocation decisions, whether there is heterogeneity by class and gender, and how these preferences translate into economic inequality. Using a novel methodological approach, we causally assessed what relative importance people attribute to different factors when distributing limited resources in a workplace setting. We thereby distinguish between need, merit, and equality as fundamental principles of distributive justice (Deutsch, 1975; Gilgen, 2022; Miller, 1992). Since each respondent distributed a fixed sum between three hypothetical employees, we further explored how perceived economic inequality shapes distributional preferences as well as the resulting inequality from these distributions. With this approach, we move beyond individualistic assessments of fairness to examine how inequality perceptions influence actual allocation behavior.

To address these questions, we developed and implemented a distributional survey experiment (DSE), first introduced by Gilgen (2022), embedded within a representative survey with about 2,000 respondents in Switzerland. In this experiment, respondents distributed CHF 18,000 among three fictitious hospital employees whose characteristics were experimentally manipulated across seven dimensions including ascriptive characteristics (gender, ethnic background), need (relationship status, children, health), and merit (occupation, dedication to job). Unlike traditional factorial survey experiments that measure fairness perceptions of given distributions, our DSE directly captures allocation decisions, allowing us to assess how different justice principles shape actual distributional choices and how they influence the resulting inequality. At the same time, the DSE has the additional quality of taking into account the inherent interdependence involved in the the distribution of limited goods among a set of people.

On the whole, we find strong evidence that merit considerations dominate workplace allocation decisions, consistent with theoretical expectations about market-based environments (Liebig et al., 2015; Narisada et al., 2021). Medical doctors received substantial premiums (approximately CHF 3,150 more than cleaners), while job dedication was rewarded with additional allocations of up to CHF 825. This pattern aligns with research emphasizing the prevalence of merit-based thinking in workplace settings (Deutsch, 1975; Fiske, 1993).

However, even in a market-pricing (Fiske, 1993) situation, merit is not the sole factor people take into consideration. Needs were also factored into people's justice evaluations, particularly regarding family responsibilities, with respondents allocating approximately CHF 300 more to employees with children. This suggests that social responsibility concerns influence distribution preferences even in a workplace setting—at least in the general population. Meanwhile, the health status of the employees was not considered relevant for the allocation of fair wages, possibly reflecting a norm that health-related needs should be addressed through separate support systems.

Additionally, we find evidence for systematic discrimination based on gender and (perceived) ethnicity. Women received approximately CHF 225 less per month, while men with Slavic or Arabic names faced penalties of CHF 170-180. These findings provide experimental evidence for discriminatory allocation patterns that mirror real-world wage gaps, extending previous factorial survey research on the fairness of earnings (Auspurg et al., 2017) by demonstrating how these biases manifest in actual distributions.

Looking at the resulting inequality of the distributions in the experiment, we find that respondents who perceived greater inequality than they deem just distribute salaries more equally in the experiment. Specifically, for each unit increase in the ratio of perceived versus desired inequality, the resulting Gini coefficient decreased by approximately 0.03 points. This finding suggests that awareness of excessive inequality may activate egalitarian preferences, consistent with theories linking inequality perceptions to distributive justice concerns and demands for reducing inequality (Huang, 2019; Petkanopoulou et al., 2025). In addition to the role of perceived inequality, respondents with higher incomes and those identifying with higher social classes consistently created more unequal distributions. These findings align with research suggesting that socio-economic position shapes attitudes toward redistribution (Fong, 2001), but extend this work by demonstrating how class position influences actual allocations/behavior and not just policy preferences.

Our findings contribute to debates in distributive justice research in several ways. First, they provide empirical support for multi-principle theories of distributive justice (Deutsch, 1975; Miller, 1992) by demonstrating that people simultaneously consider need, merit, equality as well as ascriptive characteristics when making allocation decisions (Gilgen, 2022). Second, our results speak to debates about existential versus normative standards in justice evaluations (Shepelak and Alwin, 1986). While existential standards suggest people derive fairness judgments from observed practices, our finding that perceived inequality shapes allocation decisions suggests a more complex relationship. People who perceive excessive inequality appear to reject existing distributional patterns, suggesting that normative principles can override existential acceptance of current arrangements. Third, our work extends research on the relationship between inequality perceptions and distributive justice attitudes (Heiserman and Simpson, 2021; Huang, 2019). Combining a more comprehensive assessment of perceived economic inequality with results from an experiment, we provide stronger evidence for the relationship between inequality awareness and egalitarian preferences in allocation decisions.

In addition, our study also advances the field methodologically. Using a DSE offers several advantages over predominantly used methods for studying distributive justice. Unlike laboratory experiments that often rely on convenience samples and artificial tasks, our survey experiment provides greater external validity through embeddedness in a general population survey. Additionally, the metric nature of the outcome allows for a direct quantification of the resulting inequality, moving beyond ordinal fairness ratings that dominate the literature (e.g., Alves and Rossi, 1978; Auspurg et al., 2017) to examine actual consequences on allocation decisions.

Furthermore, in contrast to factorial survey experiments, DSEs capture the interdependence inherent to distributional tasks—someone's gain is necessarily another's loss when resources are finite. This constraint forces respondents to make potentially difficult trade-offs between competing principles of justice and ascriptive characteristics of recipients, providing insights into what factors people prioritize when attempting to make just distributions of resources under conditions that come close to real-life dilemmas. Finally, we find that our key findings are robust by testing multiple inequality measures (Gini coefficient, generalized entropy measures, Theil index) and different modeling approaches, including Beta, Gamma, and Tobit regression models.

### 5.1 Limitations

Several limitations should be kept in mind when interpreting these findings. First, the hospital setting, while providing a familiar context for most respondents, represents just one organizational context. It is possible that in other workplace settings different justice norms would shape allocations. The specific professions chosen in the experiment (cleaner, nurse, doctor) also reflect particular skills and education hierarchies that might not generalize to other occupational contexts. They cover a very broad spectrum regarding the implied social status of these professions. Occupational positions, although regularly used as a proxy for unmeasured skills in wage regressions (Levenson and Zoghi, 2010), could thus possibly not only signal varying merit (in terms of invested human capital and skills) but respondents could also reward the underlying social status and prestige of these professions (principle of entitlement; Liebig et al., 2015).

Second, actual workplace compensation involves numerous factors not captured in our experiment, including seniority, performance history, market conditions, and organizational constraints. These unobserved characteristics might introduce some noise into our estimates. However, they would only bias the estimates if respondents take manipulated characteristics as heuristics for these unobserved factors in a systematic way that is also associated with the resulting distribution (Zangger, 2025).

Third, while the manipulated dimensions were chosen in accordance with the literature (Auspurg et al., 2017; Gilgen, 2022; Liebig et al., 2015), it is nevertheless possible that the resulting allocation decisions might partly also reflect alternative processes: More unequal distributions from high-status, high income respondents might also be due to ingroup favoritism, status quo rationalization or system-justifying processes (Jost et al., 2004).

Fourth, respondents in the DSE were not asked to put themselves in the position of a hospital executive when making the allocation decision, but to freely decide who should get how much according to their own ideas on what is just. While this ensures that people truly weigh the different principles of distributive justice according to their personal views of fairness, it might also underestimate the role of merit in real-world salary allocation decisions. Additionally, our study focuses on individual allocation preferences rather than group decision-making processes typical in organizational settings. Real compensation decisions often involve negotiations, committees, and institutional constraints that could soften the effects of the individual preferences and biases we observe.

Fifth, while we find significant influences of respondent level characteristics, demonstrating that people in more privileged social positions tend to distribute more unequally, the design may lack the statistical power necessary for identifying more nuanced differences between respondents. This may be the case since we only observe three outcomes per respondent. However, increasing the number of allocation decisions per respondent would also increase their cognitive load considerably. Future studies could benefit from a more explicit assessment of these trade-offs, allowing for the identification of an optimal number of vignettes to increase statistical precision while making sure the cognitive load for respondents is bearable.

Additionally, the cross-sectional design limits our ability to examine how distributional preferences might change in response to evolving inequality perceptions or changing social norms. Finally, while our Swiss sample provides important insights, awareness of cultural differences in the prevalence of justice principles (Janmaat, 2013) is important and our findings should not be generalized across (national) contexts. For example, the specific levels of perceived and desired inequality in Switzerland might not reflect patterns in societies with different inequality levels or cultural values.

### 5.2 Conclusion

Our findings have important implications for understanding wage inequality. The persistence of discriminatory allocation patterns, even in an experimental context designed to highlight fairness considerations, suggests that addressing workplace inequality requires more than appeals to fairness principles. Structural interventions that limit discretionary decision-making or require explicit justification of allocation criteria might be necessary to counteract these biases.

What is more, the role of inequality perceptions in shaping distributional preferences suggests that organizational communication about compensation policies and inequality could influence allocation decisions. On a policy level, our findings suggest that efforts to reduce inequality might benefit from increasing awareness about existing inequality levels. The tendency for those who perceive excessive inequality to distribute more equally suggests that inequality awareness could activate more egalitarian preferences among decision-makers. More generally, future research could examine how these individual preferences translate into organizational outcomes, particularly in group decision-making contexts. Additionally, since our results stem from a high-income country, cross-cultural research could examine how different societal conditions and values across different inequality levels affect attitudes on distributive justice and resulting distributions of resources.

## Data Availability

Publicly available datasets were analyzed in this study. This data can be found here https://www.swissubase.ch/de/catalogue/studies/13362/16860.
