# Supplementary material for: From principles to practice: distributive justice and the role of perceived inequality in reward allocation
Source: Front Sociol. 2025 Dec 11;10:1660806. doi: 10.3389/fsoc.2025.1660806 (PMC12738866; doi:10.3389/fsoc.2025.1660806)
Supplement: Supplementary file 1 [file Table_1.docx]

**Table A1: Descriptive statistics**

|  | | Mean | SD | min | max |
| --- | --- | --- | --- | --- | --- |
| *Vignette Characteristics* | | | | | |
| **Gender** | |  |  |  |  |
|  | Female | 0.53 | 0.50 | 0 | 1 |
|  | Male | 0.47 | 0.50 | 0 | 1 |
| **Ethnicity** | |  |  |  |  |
|  | Swiss name | 0.33 | 0.47 | 0 | 1 |
|  | Slavic name | 0.33 | 0.47 | 0 | 1 |
|  | Arabic name | 0.33 | 0.47 | 0 | 1 |
| **Partner** | |  |  |  |  |
|  | Lives with partner | 0.52 | 0.50 | 0 | 1 |
|  | Single | 0.48 | 0.50 | 0 | 1 |
| **Children** | |  |  |  |  |
|  | No children | 0.42 | 0.49 | 0 | 1 |
|  | 2 children | 0.58 | 0.49 | 0 | 1 |
| **Health** | |  |  |  |  |
|  | In poor health | 0.49 | 0.50 | 0 | 1 |
|  | Healthy | 0.51 | 0.50 | 0 | 1 |
| **Job** | |  |  |  |  |
|  | Cleaner | 0.33 | 0.47 | 0 | 1 |
|  | Nurse | 0.33 | 0.47 | 0 | 1 |
|  | Physician | 0.33 | 0.47 | 0 | 1 |
| **Dedication** | |  |  |  |  |
|  | Not very dedicated | 0.33 | 0.47 | 0 | 1 |
|  | Sometimes more, sometimes less | 0.33 | 0.47 | 0 | 1 |
|  | Very dedicated | 0.33 | 0.47 | 0 | 1 |
| **Distributed salary** | | 6010.68 | 1769.58 | 0 | 18000 |
| N | | 4863 | | | |
| *Respondent Characteristics* | | | | | |
| **Gender** | |  |  |  |  |
|  | Male | 0.52 | 0.50 | 0 | 1 |
|  | Female | 0.48 | 0.50 | 0 | 1 |
| **Migration background** | |  |  |  |  |
|  | Swiss | 0.65 | 0.48 | 0 | 1 |
|  | NW Europe & North America | 0.06 | 0.24 | 0 | 1 |
|  | Southern Europe | 0.11 | 0.31 | 0 | 1 |
|  | (South-)Eastern Europe | 0.14 | 0.35 | 0 | 1 |
|  | Rest of the world | 0.05 | 0.21 | 0 | 1 |
| **Subjective class position** | |  |  |  |  |
|  | Lower class | 0.02 | 0.15 | 0 | 1 |
|  | Working class | 0.09 | 0.29 | 0 | 1 |
|  | Lower middle class | 0.21 | 0.41 | 0 | 1 |
|  | Middle class | 0.51 | 0.50 | 0 | 1 |
|  | Upper middle class | 0.15 | 0.36 | 0 | 1 |
|  | Upper class | 0.01 | 0.11 | 0 | 1 |
| **Age** | | 50.56 | 16.15 | 19 | 96 |
| **Ratio perceived vs. desired inequality** | | 1.32 | 0.68 | 0 | 14 |
| **Personal income** | | 5691.64 | 2636.29 | 1000 | 9500 |
| **Gini** | | 0.13 | 0.07 | 0 | 1 |
| **Generalized enthropy** | | 0.04 | 0.07 | 0 | 2 |
| **Theil’s index** | | 0.04 | 0.04 | 0 | 1 |
| *N* | | 1617 | | | |
